# The Effect of Post-Transplant Cyclophosphamide Administration on Graft-Versus-Host Disease in Allogeneic Bone Marrow Transplantation

**DOI:** 10.3390/cancers18030386

**Published:** 2026-01-27

**Authors:** Selda Kahraman, Evren Ozdemir

**Affiliations:** Department of Hematology, Medicana International Izmir Hospital, 35170 Izmir, Turkey; evren.ozdemir@medicana.com.tr

**Keywords:** bone marrow transplantation, graft-versus-host disease, cyclophosphamide

## Abstract

**Aim**: In this study, we aimed to compare patients receiving PTcy with those receiving standard graft-versus-host disease (GVHD) prophylaxis in terms of GVHD development, disease relapse, overall survival, transplant-related mortality, and infection development **Methods**: Data from 78 patients who underwent allogeneic stem cell transplantation (AHSCT) at Medicana Izmir Hospital between January 2022 and June 2024 were retrospectively evaluated. **Results**: Myeloablative-related AHSCT was performed on 38 patients (48.7%), myeloablative-unrelated AHSCT was performed on 26 patients (33.3%), and haploidentical AHSCT was performed on 14 patients (17.9%). Acute GVHD was observed in 42 patients (53.8%); it was observed significantly less frequently in the group that received PTcy (*p* = 0.032) In 15 patients (19.2%), chronic GVHD developed following acute GVHD. It was found that chronic GVHD occurred more frequently in those who did not receive PTcy (*p* = 0.0001), in sibling transplants (*p* = 0.037), in those without febrile neutropenia (*p* = 0.021), and in those with high CMV-DNA levels (*p* = 0.040). The median OS (months) was found to be 79.16 months. The median OS (months) was higher in patients in the good AML cytogenetic risk group (*p* < 0.001) and in those who underwent transplantation in first remission (*p* = 0.021). **Conclusions**: PTcy significantly reduced the development of acute and chronic GVHD.

## Simple Summary

This study aimed to investigate the effect of post-transplant cyclophosphamide (PTcy) use on graft-versus-host disease in patients undergoing allogeneic stem cell transplantation. 78 patients who underwent transplantation at our hospital were included in this study. Our findings showed that in all transplantation types, the development of acute and chronic GVHD significantly less frequently in the group that received PTcy.

## 1. Introduction

Allogeneic hematopoietic stem cell transplantation is a consolidation treatment for many hematological diseases. Although significant progress has been made in HLA with increased donor sources, graft-versus-host disease (GVHD) remains a significant complication that develops after allogeneic transplantation [1,2,3].

Control of graft-versus-host disease (GVHD) is critical for the success of allogeneic hematopoietic stem-cell transplantation (HSCT). Nearly 40 years ago, the combination of methotrexate and a calcineurin inhibitor became the cornerstone for prevention of GVHD, and little has changed since then. However, despite prophylaxis, clinically significant acute GVHD, chronic GVHD, or both develop in more than half the patients undergoing allogeneic transplantation, and these conditions cause clinically significant complications and death [4,5,6,7].

Only 30% of patients have a fully matched sibling donor, while 70% require alternative donors. The increased accessibility of non-relative and haploidentical transplants has been a driving force for the use of alternative treatment regimens to reduce the development of GVHD while preserving the graft-versus-leukemia effect of the transplant.

In 1963, Berenbaum et al. [8] reported longer survival rates in mice administered a single dose of cyclophosphamide 1–3 days after skin allotransplantation. Subsequent animal experiments and clinical studies have enhanced our understanding of the immunoregulatory effects of cyclophosphamide and have created a new field for innovation and progress in transplantation.

Cyclophosphamide is an alkylating agent that has been used for many years [9]. It is oxidatively metabolized by hepatic cytochrome P450 into two potent metabolites, namely, phosphoramide mustard and acrolein, and it prevents cell division most prominently in the G1 and S phases by cross-linking DNA strands [10]. Hematopoietic stem cells rich in the enzyme aldehyde dehydrogenase, which is necessary for the conversion of the inactive metabolite of phosphoramide mustard to carboxycyclophosphamide, are resistant to cyclophosphamide. In conclusion, cyclophosphamide can be administered without disrupting engraftment following allogeneic hematopoietic cell transplantation [11]. The fundamental difference between the effect of cyclophosphamide on T cells and that of other immunosuppressive agents is its ability to induce apoptosis. Strauss et al. [12] reported that only cyclophosphamide and methotrexate induce cell death. Additionally, cyclophosphamide triggers activation-induced cell death by increasing Fas (CD95) expression. None of the other drugs affect CD95 expression.

When we examine the immunosuppressive mechanisms of post-transplant cyclophosphamide (PTcy), early-proliferating alloreactive donor and recipient T cells are selectively eliminated. Increasing the number of T-regulatory lymphocytes balances the effect of alloreactive mechanisms. Finally, the delayed but long-term intrathymic clonal deletion of anti-host T cells ensures long-term T-cell depletion [13,14].

Most of the early clinical studies on the use of PTcy for the prevention of GVHD were conducted in haploidentical stem cell transplants. At Johns Hopkins University, O’Donnell et al. [15] published the first study on the use of cyclophosphamide in haploidentical stem cell transplantation. In the initial patients of this study, a single dose of 50 mg/kg/day cyclophosphamide was administered on the 3rd day post-transplantation. Subsequently, due to the high rate of graft rejection, a second dose of cyclophosphamide was added to the treatment regimen.

Later, Munchel et al. at Johns Hopkins University [16] reported the results of a large phase II study involving 210 patients with hematologic malignancies. Haploidentical stem cell transplantation was performed on patients using the group’s original non-myeloablative conditioning regimen and GVHD prophylaxis protocol. Permanent engraftment occurred in 87% of the patients. The incidence of 5-year transplant-related mortality (TRM) was 18%. The incidence of disease relapse was 55%, while the disease-free survival (DFS) and overall survival (OS) rates were 35% and 27%, respectively. The incidence of acute grades II to IV graft-versus-host disease (GVHD) and chronic GVHD was reported to be 27% and 13%, respectively. This also indicates that the incidence of chronic GVHD is remarkably low.

Following these studies, Bashey et al. [17] published a retrospective analysis comparing 53 patients who underwent haploidentical AHSCT with PTcy to 117 fully matched related donor recipients and 101 fully matched unrelated donor recipients who received standard GVHD prophylaxis at the same center. No difference was found among the three groups in terms of the incidence of GVHD, transplant-related mortality (TRM), relapse, and survival rates.

Over the last half-century, high-dose cyclophosphamide has been used to preserve the hematopoietic stem system and limit GVHD in post-transplant care [18,19,20]. The acceptable incidence of GVHD, particularly severe chronic GVHD, was subsequently confirmed in multicenter trials, including those involving patients receiving HLA-mismatched, unrelated donor transplants [21,22,23,24,25,26,27].

In light of these studies, we retrospectively evaluated the data of patients who underwent AHSCT at our center between January 2022 and June 2024. We aimed to compare patients receiving PTcy with those receiving standard GVHD prophylaxis in terms of GVHD development, disease relapse, DFS, OAS, TRM, and infection development.

## 2. Materials and Methods

This study was a retrospective evaluation of data from 78 patients who underwent allogeneic hematopoietic stem cell transplantation at Medicana Izmir Hospital, Izmir, Türkiye between January 2022 and June 2024.

In cases of hospital admission for transplantation, occurrences of neutropenic fever, the subsequent development of acute and chronic GVHD and CMV infections, OAS, and EFS were evaluated.

Myeloablative-related AHSCT was performed on 38 patients (48.7%), myeloablative-unrelated AHSCT was performed on 26 patients (33.3%), and haploidentical AHSCT was performed on 14 patients (17.9%).

Patient diagnoses were as follows: 37 patients (45.9%) had AML, 13 patients (9%) had MDS-RAEB1-2, 12 patients (14.2%) had ALL, 7 patients (10.3%) had multiple myeloma, 4 patients had lymphoma, 2 patients had myelofibrosis, 1 patient had aplastic anemia, 1 patient had sickle cell anemia, and 1 patient had CLL. In total, 68 patients (87%) underwent transplantation in remission, 6 patients underwent transplantation in active disease, and 4 patients underwent transplantation in partial response. No comorbidities were present in 50 patients (64.1%). The most common chronic diseases were HT and DM.

When examining the transplantation preparation regimens, it was found that fludarabine–melphalan was administered to 69 patients (88.5%), and treosulfan–melphalan treatment was given to 9 patients (11.5%). ATG was administered to 27 patients (34.6%) as part of the preparatory regimen (26 patients with unrelated donor transplants and 1 patient with related donor transplants). Furthermore, 49 patients (62.8%: 14 patients with haploidentical transplants, 26 patients with unrelated donor transplants, and 9 patients with related donor transplants) received post-transplant cyclophosphamide treatment, while 29 patients (37.2%: all with related donor transplants) did not.

In 72 transplants (92.3%), sufficient numbers of stem cells were collected from the donor in a single apheresis for the transplant; in the remaining cases, stem cells could only be collected from 5 donors over two days and from 1 donor over three days. Allogeneic stem cell transplantation was performed in 44 patients with their siblings as donors (37 fully matched and 7 haploidentical), in 4 patients with their children as donors (1 fully matched and 3 haploidentical), in 3 patients with their mothers as donors (haploidentical), in 1 patient with their father as a donor (haploidentical), and in 26 patients with unrelated donors registered with the Turkkok bone marrow bank.

All patients were started on cyclosporine and mycophenolate mofetil on day −1 for GVHD prophylaxis. Cyclophosphamide was administered as an intravenous infusion of 50 mg/kg/day on days 3 and 5 post-transplant. On day + 1 post-transplant, all patients were prophylactically started on levofloxacin flk 500 mg 1 × 1, fluconazole 200 mg IV (6 patients received voriconazole as secondary prophylaxis), and acyclovir IV treatment. When patients’ temperatures were >38 degrees, catheter–peripheral blood cultures were taken, and HRCT (high-resolution computed tomography) was performed.

Acute and chronic GVHD (graft-versus-host disease) was graded according to the IBMTR (International Bone Marrow Transplantation Registry) grading system.

### Statistical Analysis

Statistical analyses were conducted using “IBM SPSS Statistics for Windows, Version 25.0 (Statistical Package for the Social Sciences, IBM Corp., Armonk, NY, USA)”. Descriptive statistics are presented as n and % for categorical variables, and as mean ± SD and median (min–max) for continuous variables. An ROC curve analysis was used in the prediction of morbidity via various indices. The Kaplan–Meier method was used to compare survival and EFS durations among clinical groups, with *p* < 0.05 considered statistically significant.

## 3. Results

The data of 78 patients who underwent AHCST at Medicana Izmir Hospital between January 2022 and June 2024 were retrospectively evaluated.

Seventy-eight patients who underwent AHSCT at our center were included in this study. Of these, 36 (46.2%) were female, and 42 (53.8%) were male. Myeloablative-related AHSCT was performed in 38 patients (48.7%), myeloablative-unrelated AHSCT was performed in 26 patients (33.3%), and haploidentical AHSCT was performed in 14 patients (17.9%).

Acute GVHD (acute graft-versus-host disease): According to the IBMTR (International Bone Marrow Transplant Registry) grading system, acute GVHD was not observed in 36 patients (46.2%), while grade A was observed in 20 patients (25.6%), grade B was observed in 9 patients (11.5%), grade C was observed in 3 patients (3.8%), and grade D was observed in 10 patients (12.8%).

Regarding the development of acute GVHD post-transplant, skin GVHD was not observed in 39 patients (50%), while grade 1 was observed in 27 patients (34.6%), grade 2 was observed in 10 patients (12.8%), and grade 3 was observed in 2 patients (2.6%).

Gastrointestinal system (GIS) GVHD was not observed in 58 patients (75.3%), while grade 1 was observed in 5 patients (6.5%), grade 2 was observed in 2 patients (2.6%), grade 3 was observed in 5 patients (6.5%), and grade 4 was observed in 7 patients (9.1%).

Liver GVHD was not observed in 68 patients (87.2%), while grade 1 was observed in 5 patients (6.4%), grade 2 was observed in 4 patients (5.1%), and grade 4 was observed in 1 patient (1.3%).

A total of 19 patients (24.4%) developed only skin GVHD, 1 patient (1.3%) developed GIS GVHD, 1 patient (1.3%) developed liver GVHD, 13 patients (16.7%) developed skin and GIS GVHD, 3 patients (3.8%) developed skin and liver GVHD, 4 patients (5.1%) developed skin–GIS–liver GVHD, and 1 patient (1.3%) developed GIS and liver GVHD.

All patients received cyclosporine and mycophenolate mofetil treatment for GVHD prophylaxis. When examining the immunosuppressive therapies received by patients who developed acute GVHD, it was found that 28 patients received cyclosporine, mycophenolate mofetil, and methylprednisolone; 8 patients received tacrolimus, mycophenolate mofetil, methylprednisolone, and ruxolitinib; 2 patients received tacrolimus, mycophenolate mofetil, methylprednisolone, cyclophosphamide, ruxolitinib, and prolastin C; and 4 patients received cyclosporine, mycophenolate mofetil, methylprednisolone, cyclophosphamide, ruxolitinib, and mesenchymal stem cells.

Steroid resistance was observed in 14 patients with acute GVHD, and other immunosuppressive treatments were started.

As shown in Table 1, regarding the clinical and laboratory variables affecting acute GVHD, only ferritin (*p* = 0.016) was found to be significantly lower in the group with acute GVHD, and acute GVHD was observed significantly less frequently in the group that received PTcy (*p* = 0.032).

A total of 63 patients (80.8%) did not develop chronic GVHD, while 15 patients (19.2%) developed it following acute GVHD. A total of 12 patients (15.4%) developed chronic GVHD with skin involvement, 11 patients (14.1%) developed chronic GVHD with eye involvement, 12 patients (15.4%) developed chronic GVHD with mouth involvement, 3 patients (3.8%) developed chronic GVHD with esophageal involvement, 1 patient (1.3%) developed chronic GVHD with serous membrane involvement, 3 patients (3.8%) developed chronic GVHD with liver involvement, 3 patients (3.8%) developed chronic GVHD with bronchiolitis obliterans involvement, and 6 patients (7.7%) developed chronic GVHD with joint fascia involvement.

As shown in Table 2, regarding the clinical and laboratory variables affecting chronic GVHD, it was found that chronic GVHD occurred more frequently in those who did not receive PTcy (*p* = 0.0001), in sibling transplants (*p* = 0.037), in those without febrile neutropenia (*p* = 0.021), and in those with high CMV-DNA levels (*p* = 0.040).

When all AML-MDS patients (N = 50, 64.1%) who received PTcy were examined regardless of the type of transplant, the rates of developing acute and chronic GVHD were found to be significantly lower (*p* = 0.036–0.001, respectively).

Regarding other diseases, 8 out of 12 ALL patients received PTcy, 6 patients developed acute GVHD (3 received cyclophosphamide, and 3 did not), and 2 patients developed chronic GVHD (neither received cyclophosphamide). No significant difference was observed in acute and chronic GVHD among patients who had ALL and received PTcy (*p* = 0.54–0.41, respectively).

Of the 16 patients with other diagnoses and multiple myeloma, 8 received PTcy, and 8 developed acute GVHD (4 received cyclophosphamide, and 4 did not). Chronic GVHD was observed in only 1 patient who did not receive PTcy. Chronic GVHD was statistically significantly less common in this group (*p* < 0.05).

As shown in Table 3, the duration of platelet engraftment (*p* = 0.046) was found to be statistically significantly associated with the occurrence of mortality.

As shown in Table 4, the median OS (months) was 79.16 months (95% CI: 24.47–133.85). The median OS (months) was found to be statistically significantly higher in patients with a favorable AML cytogenetic risk profile (*p* < 0.001) and in those undergoing transplantation in first remission (*p* = 0.021).

Age, gender, type of transplantation, disease, preparative regimen for transplantation, antifungal usage, and development of CMV infection did not affect OAS (*p* > 0.05). As shown in Table 5, the median EFS (months) was not reached.

Table 6 shows that the median OS (months) was not reached in patients diagnosed with AML. As the cytogenetic risk category increased in patients with AML (*p* < 0.001) and in those with steroid resistance in acute GVHD (*p* = 0.026), the median OS (months) was found to be significantly shorter.

As shown in Table 7, the median OS (months) of patients diagnosed with ALL was 13.66 (95% CI: -). In females (*p* = 0.036) and in patients receiving ATG (*p* = 0.003), the median OS (months) was found to be significantly higher.

During transplantation, 55 patients (70.5%) developed febrile neutropenia, while 23 patients (29.5%) did not. Ground-glass opacities were detected in 10 patients, nodular lesions with surrounding halo signs were detected in 5 patients, and lobar pneumonia was detected in 2 patients on HRCT scans obtained during febrile neutropenia. The HRCT scans of 38 patients were normal.

At the time of transplantation, 72 patients (92.3%) and 6 patients (7.7%) were receiving fluconazole or voriconazole prophylaxis, respectively. During the transplant-associated neutropenic fever, 31 patients received empirical antifungal therapy, 8 patients received preemptive therapy, and 2 patients received proven antifungal treatment.

During treatment, 19 patients received meropenem and teicoplanin; 16 patients received meropenem, teicoplanin, and amikacin; 10 patients received meropenem, tigecycline, and amikacin; 10 patients received meropenem, colistin, tigecycline, and amikacin; and 1 patient received ceftazidime–avibactam, tigecycline, colistin, and amikacin.

In cultures obtained during the febrile period, 7 patients had ESBL-negative *E. coli* 6 patients had methicillin-resistant coagulase-negative *Staphylococcus aureus* (MRCNS), 5 patients had *Staphylococcus epidermidis*, 3 patients had carbapenem-sensitive bacteria, 1 patient had carbapenem-resistant *Klebsiella pneumoniae*, 3 patients had *Staphylococcus hominis*, 1 patient had *Pseudomonas aeruginosa*, 1 patient had *Enterococcus faecium*, 1 patient had *Staphylococcus haemolyticus*, and 1 patient had *Candida parapsilosis*, while no growth was observed in the blood cultures of 27 patients. *E. coli* was detected in the urine cultures of 4 patients, carbapenem-resistant *Klebsiella pneumoniae* in 1 patient, carbapenem-sensitive *Klebsiella pneumoniae* in 1 patient, and *Stenotrophomonas maltophilia* in 1 patient, while no growth was observed in the urine cultures of 49 patients. In sputum cultures, *Candida glabrata* was isolated in 2 patients, *Aspergillus fumigatus* in 1 patient, *Enterococcus faecium* in 1 patient, and *E. coli* in 1 patient.

Primary engraftment failure was observed in one patient and secondary engraftment failure was observed in five patients following transplantation (7.8% in total). Only two patients were lost within the first 30 days of transplantation (2.6%). During the first 100 days post-transplant, 12 patients died, and during the first year, 23 patients died.

It was found that 25 patients died by the last follow-up date due to various reasons. The causes of death are presented in Table 8. During the follow-up, 7 patients experienced relapse, and 5 patients were lost due to disease progression, while non-relapse mortality occurred in 20 patients.

## 4. Discussion

The administration of cyclophosphamide (PTCy) following allogeneic stem cell transplantation contributes to the development of peripheral tolerance by eliminating alloreactive donor T cells through clonal deletion and Treg suppression. Recent studies have shown that alloreactive T cells are not completely eliminated, their proliferation is reduced, and their functions are impaired [14,28,29].

The first study on post-transplant cyclophosphamide administration was published by O’Donnell et al. at Johns Hopkins University. Patients who underwent haploidentical stem cell transplantation were treated with cyclophosphamide post-transplantation [15]. The use of PTCy led to a significant transformation by expanding haploidentical transplantation practices.

Subsequently, Luznik et al. published a study in which they performed haploidentical stem cell transplantation in 68 patients with advanced hematologic malignancies following a non-myeloablative conditioning regimen. On the 3rd and/or 4th day post-transplant, cyclophosphamide treatment was given to 28 patients for 1 day and to 40 patients for 2 days; all patients received mycophenolate mofetil and tacrolimus for GVHD prophylaxis. Engraftment failure developed in nine patients (13%), while the median day of neutrophil engraftment was day 15 and that of platelet engraftment was day 24. Transplant-related mortality was 15%, the one-year relapse rate was 51%, two-year overall survival was 36%, two-year event-free survival was 26%, grade 2–4 acute GVHD was observed in 34% of patients, and grade 3–4 acute GVHD was observed in 6% of patients. Regarding chronic GVHD, it was observed in 25% of patients who received a single dose of cyclophosphamide, whereas it was observed in only 5% of patients who received a double dose of cyclophosphamide [28].

Following this success achieved in haploidentical transplantation, PTCy-based GVHD prophylaxis regimens have been tested as alternatives to traditional calcineurin inhibitor (CNI)-based regimens and have begun to be administered in fully matched related donor (MRD) and fully matched unrelated donor (MUD) settings.

Luznik et al. at Johns Hopkins University initiated PTcy administration in fully matched related and unrelated transplants. They conducted a study in which they administered 50 mg/kg/day cyclophosphamide treatment on days 3–4 post-transplant to 78 fully matched related and 39 fully matched unrelated transplant patients following a myeloablative conditioning regimen; 63% of fully matched transplant patients and 54% of unrelated transplant patients were in remission at the time of transplantation. Transplant-related mortality was observed in 17% of patients, while the 2-year OAS was found to be 55%, and EFS was 39%. Acute grade 2–4 GVHD was observed in 43% of patients, while chronic GVHD was observed in 10% of patients [30].

Our study included 78 patients. Myeloablative-related allogeneic hematopoietic stem cell transplantation was performed in 38 patients (48.7%), myeloablative-unrelated allogeneic hematopoietic stem cell transplantation was performed in 26 patients (33.3%), and haploidentical allogeneic hematopoietic stem cell transplantation was performed in 14 patients (17.9%). Acute GVHD was observed in 53.8% of patients (N: 42 patients; 22 received PTcy, and 20 did not), with grades 2–4 observed in 22 patients (28.1%) and grades 3–4 observed in 13 patients (16.6%).

Acute GVHD was not observed in 36 patients (27 received PTcy, and 9 did not). Steroid resistance was observed in 14 patients with acute GVHD, and other immunosuppressive treatments were started.

In our study, only ferritin (*p* = 0.016) was found to be significantly lower in the group with acute GVHD, and acute GVHD was observed significantly less frequently in the group that received post-transplant cyclophosphamide (*p* = 0.032) (Table 1).

Chronic GVHD was observed in 14 patients (17.94%) (11 received PTcy, and 3 did not), whereas it was not observed in 64 patients (36 received PTcy, and 18 did not).

Regarding the clinical and laboratory variables affecting chronic GVHD, it was found that chronic GVHD occurred more frequently in those who did not receive post-transplant cyclophosphamide (*p* = 0.0001), in sibling transplants (*p* = 0.037), and in those with high CMV-DNA levels (*p* = 0.040). In sibling transplants, chronic GVHD occurred more frequently because post-transplant cyclophosphamide was not administered (Table 2).

In other studies, the groups were homogeneous, and all patients were given PTcy, whereas in our study, some of the patients also received cyclophosphamide. Despite this, our acute and chronic GVHD rates improved to levels similar to those observed in other studies.

In another clinical study conducted by Kanarky et al., 45 fully matched related and 47 fully matched unrelated hematological malignancy patients received cyclophosphamide treatment for 2 days post-transplant following a myeloablative conditioning regimen containing fludarabine and busulfan. Twenty-five patients underwent transplantation with active disease. In the median 1.6-year follow-up, TRM was found to be 16%, OAS was 62%, and EFS was 67%. Acute grade 2–4 GVHD was observed in 51% of patients, acute grade 3–4 GVHD was observed in 15% of patients, and chronic GVHD was observed in 14% of patients [31].

In a large-scale retrospective registry study based on CIBMTR data, the use of PTCy was compared between 284 adults undergoing matched unrelated donor (MUD) transplantation and 2036 adult patients undergoing haploidentical transplantation. It demonstrated that higher grades (III-V) of acute GVHD and higher rates of chronic GVHD are observed in haploidentical transplants and that MUD transplantation still remains the gold standard in this context [32].

Finally, another study involving 431 leukemia patients compared the combination of PTCy + tacrolimus + mycophenolate mofetil (MMF) with the standard tacrolimus-MTX prophylaxis regimen. Among patients undergoing reduced-intensity or non-myeloablative 8/8 or 7/8 matched related donor HSCT, the incidence of grade III-IV aGVHD was found to be lower in the PTCy group (6.3%) than in the standard prophylaxis group (14.7%). Similarly, the 12-month incidence of cGVHD was lower in the PTCy group (21.9%) than in the standard prophylaxis group (35.1%) [33].

The median OS of our patients was determined to be 79.16 months (95% CI: 24.47–133.85 months). The median OS (months) was found to be statistically significantly higher in patients with a favorable AML cytogenetic risk profile (*p* < 0.001) and in those undergoing transplant in the first remission (*p* = 0.021).

Age, gender, type of transplantation, disease, preparative regimen for transplantation, PTcy usage, antifungal usage, and development of CMV infection did not affect OAS (*p* > 0.05). The median EFS (months) was not reached.

As the cytogenetic risk category increased in patients with AML (*p* < 0.001) and in those with steroid resistance in acute GVHD (*p* = 0.026), the median OS (months) was found to be significantly shorter.

The median OS (months) of patients diagnosed with ALL was determined to be 13.66 months (95% CI: -). The median OS (months) was found to be significantly higher in women (*p* = 0.036) and in patients receiving ATG (*p* = 0.003). The overall 2-year OAS was determined to be 67.6%, and the 5-year OAS was 56.9%, with a median of 79.16 months (range of 24.47–133.85 months).

## 5. Conclusions

Post-transplant cyclophosphamide (PTCy) is a significant immunomodulatory strategy in allogeneic hematopoietic stem cell transplantation that supports the development of tolerance by reducing the incidence of GVHD. Current clinical studies demonstrate that PTCy offers an alternative to traditional GVHD prophylaxis regimens by showcasing its effectiveness in haploidentical and fully matched transplantation.

However, the use of PTCy needs to be evaluated with more comprehensive prospective studies where the disease types and patient numbers are homogeneous; these studies will elucidate whether the effect of PTcy on GVHD is specific to a particular type of cancer or is a general effect that is independent of disease type.

## Figures and Tables

**Table 1:** Comparison of various clinical variables that may affect the development of acute GVHD.

Variable	Acute GVHD		*p*
	None N = 36	Present N = 42	
Received ATG, n (%)			
Did not receive	20 (55.6)	31 (73.8)	0.091
Received	16 (44.4)	11 (26.2)	
Post-transplant cycle, n (%)			
Did not receive	9 (25)	20 (47.6)	0.032 ^b^
Received	27 (75)	22 (52.4)	
Conditioning regimen, n (%)			
Fludara–melphalan	29 (80.6)	39 (92.9)	0.111 ^b^
Fludara–treosulfan	6 (16.7)	2 (4.8)	
Treosulfan + melphalan	1 (2.8)	0 (0)	
Fludara + melphalan + IVIG	0 (0)	1 (2.4)	
Compatibility, median (IQR)	100.0 (7.50)	100.0 (10.0)	0.793 ^c^
Stem cell count, median (IQR)	6.7 (3.5)	7.5 (2.9)	0.113 ^c^
Donor relation, n (%)			
Sibling	17 (47.2)	26 (61.9)	0.154 ^b^
Mother	0 (0)	3 (7.1)	
Child	2 (5.6)	2 (4.8)	
Unrelated	16 (44.4)	11 (26.2)	
Father	1 (2.8)	0 (0)	
Donor age	43.5 (20–73)	44.5 (20–72)	0.437 ^c^
Female donor birth	1.0 (0–3)	2.0 (0–8)	0.405 ^c^
Donor sex, n (%)			
Female patient–female donor	5 (13.9)	7 (16.7)	0.798 ^a^
Female patient–male donor	10 (27.8)	13 (31)	
Male patient–male donor	15 (41.7)	13 (31)	
Male patient–female donor	6 (16.7)	9 (21.4)	
Blood group, n (%)			
Identical	19 (52.8)	25 (59.5)	0.082 ^b^
ABO-incompatible	11 (30.6)	15 (35.7)	
Rh-incompatible	1 (2.8)	2 (4.8)	
ABO-Rh-incompatible	5 (13.9)	0 (0)	
Ferritin, median (IQR)	2183.4 (1680.2)	1277.0 (1992.5)	0.016 ^c^
Febrile neutropenia, n (%)			
None	7 (19.4)	16 (38.1)	0.072 ^a^
Present	29 (80.6)	26 (61.9)	
CMV DNA, median (IQR)	1161.0 (6822.2)	1584.0 (8320.2)	0.203 ^c^
Defibrotide use, n (%)			
Did not receive	16 (44.4)	24 (57.1)	0.263 ^a^
Prophylaxis	20 (55.6)	18 (42.9)	

^a^: Pearson chi-square test, ^b^: Fisher’s exact test, ^c^: Mann–Whitney U test; *p* < 0.05 indicates statistical significance.

**Table 2:** Comparison of various clinical variables that may affect the development of chronic GVHD.

Variable	Chronic GVHD		
	None N = 64	Present N = 14	*p*
Received ATG, n (%)			
Did not receive	38 (59.4)	13 (92.9)	0.091
Received	26 (40.6)	1 (7.1)	
Post-transplant cycle, n (%)			
Did not receive	18 (28.1)	11 (78.6)	0.0001 ^b^
Received	36 (71.9)	3 (21.4)	
Conditioning regimen, n (%)			
Fludara–melphalan	57 (89.1)	11 (78.6)	0.238 ^b^
Fludara–treosulfan	6 (9.4)	2 (14.3)	
Treosulfan + melphalan	1 (1.6)	0 (0)	
Fludara + melphalan + IVIG	0 (0)	1 (7.1)	
Compatibility, median (IQR)	100.0 (10.0)	100.0 (0.0)	0.070 ^c^
Stem cell count, median (IQR)	6.9 (3.4)	8.1 (2.3)	0.247 ^c^
Donor relation, n (%)			
Sibling	30 (46.9)	13 (92.9)	0.037 ^b^
Mother	3 (4.7)	0 (0)	
Child	4 (6.3)	0 (0)	
Unrelated	26 (40.6)	1 (7.1)	
Father	1 (1.6)	0 (0)	
Donor age	44.0 (20–72)	44.0 (24–73)	0.840 ^c^
Female donor birth	1.5 (0–4)	2.0 (0–8)	0.231 ^c^
Donor sex, n (%)			
Female patient–female donor	9 (14.1)	3 (21.4)	0.420 ^b^
Female patient–male donor	21 (32.8)	2 (14.3)	
Male patient–male donor	21 (32.8)	7 (50)	
Male patient–female donor	13 (20.3)	2 (14.3)	
Blood group, n (%)			
Identical	38 (59.4)	6 (42.9)	0.226 ^b^
ABO-incompatible	18 (28.1)	8 (57.1)	
Rh-incompatible	3 (4.7)	0 (0)	
ABO-Rh-incompatible	5 (7.8)	0 (0)	
Ferritin, median (IQR)	1745.7 (2008.2)	1042.6 (2074.8)	0.143 ^c^
Febrile neutropenia, n (%)			
None	15 (23.4)	8 (57.1)	0.021
Present	49 (76.6)	6 (42.9)	
CMV DNA, median (IQR)	390.5 (2855.5)	1618 (7832)	0.040 ^c^
Defibrotide use, n (%)			
Did not receive	31 (48.4)	9 (64.3)	0.283 ^a^
Prophylaxis	33 (51.6)	5 (35.7)	

^a^: Pearson chi-square test, ^b^: Fisher’s exact test, ^c^: Mann–Whitney U test; *p* < 0.05 indicates statistical significance.

**Table 3:** Analysis of various parameter values in predicting mortality.

Variable	AUC	95% CI	Cut-Off	Sensitivity (%)	Specificity (%)	*p*
Platelet engraftment	0.650	0.505–0.796	≥13.50	52.4	65.4	0.046

AUC, area under the curve; 95% CI, confidence interval.

**Table 4:** Comparisons of OS of patients (N = 78).

OS (Months)	2-Year (%)	5-Year (%)	Median (95% CI)	*p*
Overall	67.6	56.9	79.16 (24.47–133.85)	
Age				
≤60	69.5	55.3	79.16 (39.22–119.11)	0.945
>60	61.7	61.7	114.50 (-)	
Sex				
Female	63.0	48.5	53.46 (0.00–110.60)	0.186
Male	70.5	70.5	114.50 (-)	
Type of transplant				
Myeloablative related	68.5	50.3	106.33 (18.90–193.75)	0.603
Unrelated	59.7	59.7	59.7 (21.5–182.28)	
Haploidentical	82.1	82.1	79.16 (0.00–173.23)	
Diagnosis				
AML	65.3	65.3	- (-)	0.162
ALL	49.9	-	13.66 (-)	
Other	86.2	67.1	106.33 (42.29–170.37)	
AML risk				
Good	-	-	6.70 (-)	<0.001
Moderate	-	-	12.66 (12.61–12.72)	
Poor	69.4	69.4	- (-)	
Remission no.				
1	54.2	-	11.65 (8.79–54.47)	0.021
2	84.6	84.6	106.33 (-)	
3 or above	100.0	80.0	114.50 (-)	
Platelet engraftment				
<13.50	78.8	47.3	53.46 (11.25–95.67)	0.894
≥13.50	64.7	64.7	114.50 (12.45–216.55)	

Kaplan–Meier curve, log-rank test; *p* < 0.05 indicates statistical significance.

**Table 5:** Comparisons of EFS of patients (N = 78).

EFS (Months)	2-Year (%)	5-Year (%)	Median (95% CI)	*p*
Overall	83.1	83.1	- (-)	
Sex				
Female	72.4	72.4	- (-)	0.770
Male	87.4	-	- (-)	
Diagnosis				
AML	92.9		- (-)	0.417
ALL	70.0	-	- (-)	
Other	75.0	75.0	- (-)	

Kaplan–Meier curve, log-rank test; *p* < 0.05 indicates statistical significance.

**Table 6:** Comparisons of OS of AML patients (N = 50).

OS (Months)	2-Year (%)	5-Year (%)	Median (95% CI)	*p*
Overall	65.3	65.3	- (-)	
Age				
≤60	69.9	69.9	- (-)	0.751
>60	53.3	-	- (-)	
Sex				
Female	56.0	56.0	- (-)	0.141
Male	72.5	72.5	- (-)	
Type of transplant				
Myeloablative related	51.9	-	- (-)	0.755
Unrelated	69.3	69.3	- (-)	
Haploidentical	75.0	75.0	- (-)	
AML risk				
Good	-	-	6.70 (-)	<0.001
Moderate	-	-	12.66 (12.61–12.72)	
Poor	69.4	69.4	- (-)	
Acute GVHD steroid resistance				
None	76.5	76.5	- (-)	0.026
Present	33.3	-	12.66 (0.00–27.55)	

Kaplan–Meier curve, log-rank test; *p* < 0.05 indicates statistical significance.

**Table 7:** Comparisons of OS of ALL patients (N = 11).

OS (Months)	2-Year (%)	Median (95% CI)	*p*
Overall	49.9	13.66 (-)	
Age			
≤60	56.0	- (-)	0.376
>60	-	11.60 (-)	
Sex			
Female	-	7.93 (5.69–10.17)	0.036
Male	60.0	- (-)	
ATG			
Did not receive	70.0	- (-)	0.003
Received	-	7.93 (5.69–10.17)	

Kaplan–Meier curve, log-rank test; *p* < 0.05 indicates statistical significance, ATG: Anti-Thymocyte Globulin.

**Table 8:** Causes of patient mortality.

Cause of Death	Patient Count	Percentage (%)
Disease progression–pneumonia	5	6.4
Pneumonia	4	5.1
GVHD	4	5.1
Pneumonia–GVHD	4	5.1
*Klebsiella* pneumonia sepsis	2	2.6
*Pseudomonas aeruginosa* sepsis	2	2.6
Engraftment failure–pneumonia	1	1.3
Multiple organ failure	2	2.6
COVID-19 pneumonia	1	1.3

## Data Availability

The original contributions presented in this study are included in the article. Further inquiries can be directed to the corresponding author.
